# Dynamics of Nanoparticle-Protein Corona Complex Formation: Analytical Results from Population Balance Equations

**DOI:** 10.1371/journal.pone.0064690

**Published:** 2013-05-31

**Authors:** Faryad Darabi Sahneh, Caterina Scoglio, Jim Riviere

**Affiliations:** 1 Institute of Computational Comparative Medicine (ICCM), Kansas State University, Manhattan, Kansas, United States of America; 2 Electrical & Computer Engineering, Kansas State University, Manhattan, Kansas, United States of America; 3 Anatomy & Physiology, Kansas State University, Manhattan, Kansas, United States of America; Jacobs University Bremen, Germany

## Abstract

**Background:**

Nanoparticle-protein corona complex formation involves absorption of protein molecules onto nanoparticle surfaces in a physiological environment. Understanding the corona formation process is crucial in predicting nanoparticle behavior in biological systems, including applications of nanotoxicology and development of nano drug delivery platforms.

**Method:**

This paper extends the modeling work in to derive a mathematical model describing the dynamics of nanoparticle corona complex formation from population balance equations. We apply nonlinear dynamics techniques to derive analytical results for the composition of nanoparticle-protein corona complex, and validate our results through numerical simulations.

**Results:**

The model presented in this paper exhibits two phases of corona complex dynamics. In the first phase, proteins rapidly bind to the free surface of nanoparticles, leading to a *metastable composition*. During the second phase, continuous association and dissociation of protein molecules with nanoparticles slowly changes the composition of the corona complex. Given sufficient time, composition of the corona complex reaches an equilibrium state of *stable composition*. We find analytical approximate formulae for metastable and stable compositions of corona complex. Our formulae are very well-structured to clearly identify important parameters determining corona composition.

**Conclusion:**

The dynamics of biocorona formation constitute vital aspect of interactions between nanoparticles and living organisms. Our results further understanding of these dynamics through quantitation of experimental conditions, modeling results for *in vitro* systems to better predict behavior for *in vivo* systems. One potential application would involve a single cell culture medium related to a complex protein medium, such as blood or tissue fluid.

## Introduction

Corona complex composition greatly influences the ability of nanoparticles to deliver drugs to specific receptors, as well as to modulate their toxicity. Numerous interactions occur when nanoparticles are introduced to a biological fluid. Proteins compete with other biomolecules to surround nanoparticles, forming protein coronae, thus defining the biological fingerprint of the particles [2]–[4]. Corona formation is complex, contingent upon protein molecule type, size, and conformational flexibility, nanoparticle type, size, shape, electric charge, and hydrophobicity, as well as medium-related factors (e.g., pH and ionic strength) [2], [5], [6]. Recent comprehensive surveys present information about the nanoparticle corona formation process ([3], [6]). Quantitation and prediction of corona formation is vital for standardized safe medical use of nanoparticles.

Quantification of protein and nanoparticle interaction is attracting substantial attention at the cutting edge of research (see e.g., [4], [7], [8]). The dynamics of the corona complex formation process are very challenging to study due to inherent complexity, rendering extremely discrete experimental results. Moreover, quantitative approaches cannot capture all involved complexities for the real word situation is often too complex and variable to address. Models have the unique ability to provide insight into specific aspects of the process that, in an experimental context, are too difficult to isolate and extract. Vilaseca *et al.* [9] recently simulated molecular dynamics (MD) to study the surface-adsorption of proteins. They reduced the complexity of a full modeling by approximating protein molecules as single, rigid entities. Kinetic modeling of corona complex formation process dramatically decreases computational cost, though adopting several simplifying assumptions. Significant contributions include Dobay *et al.* [10] use of stochastic process algebra to study the evolution and subcellular distribution of nanoparticles in living cells. Mathematical modeling helps us learn principles and develop quantitative approaches that cannot be experimentally extracted. Most importantly, simpler mathematical models provide quantitative endpoints against which experiments can be designed and evaluated.

Dell'Orco *et al.* [1] proposed the first simple dynamical model of corona formation where a set of ordinary differential equations represent the time variations in corona composition. Recently, they extended their model to assess the delivery success rate of nanoparticles forming protein corona complex [11]. They consider three proteins: human serum albumin (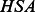), high density lipoprotein (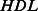), and fibrinogen (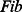), with experimentally determined association and dissociation rates. A very interesting observation is the existence of two equilibrium points: a first *metastable equilibrium* quickly reached after nanoparticles are injected in the fluid, and a final *stable equilibrium* point reached after a much longer time interval [1]. However, this numerical observation is not explained analytically.

In this paper, the model developed in [1] is extended to provide analytical results describing the two equilibrium points in terms of both corona composition and process time constant. Additionally, we develop a reduced complexity model separating fast and slow dynamics of biocorona formation process. We deduce the metastable composition point is largely determined by association rates weighted by corresponding initial protein concentrations and the stable equilibrium point is determined by equilibrium constants weighted by corresponding initial protein concentrations. Moreover, we find metastable equilibrium exhibits a time constant of the order of association rates inverses weighted by initial protein concentrations, while the stable equilibrium has a time constant of the order of dissociation rate inverses. Overall, our results extend and simplify the use of the Dell'Orco *et al.* model in [1]. In brief, the main contributions of this paper are:

- Descriptive biochemical equations (1–3) representing the fundamental mechanisms influencing corona formation dynamics.
- Explicit analytical formulae for metastable composition (see (15)) and stable composition (see (17)), simplified to identify key factors of corona complex formation.
- Reduced complexity model for corona formation dynamics (see (18)) dramatically decreasing numerical simulation run time, effectively improving stability of numerical simulation.
- Sensitivity analysis of metastable and stable corona compositions respective to association and dissociation rates uncertainties (see (20–21)).

## Results

As nanoparticles come in contact with physiological fluid, they are engulfed by different types of biomolecules. A single layer of biomolecules tightly binds nanoparticle surfaces, forming the ‘hard corona.’ Additional dynamic layers of biomolecules loosely attach to the hard corona, forming the ‘soft corona’ [5], [12], [13]. While the hard corona exhibits stable composition, the biomolecules composing the hard corona alter when nanoparticles move from one environment to another [14], [15]. Even when nanoparticles remain in the same environment, hard corona formation involves a transient state where biomolecule exchange can last for hours [12], [16].

Below, we develop a dynamical model for hard corona complex formation from corresponding population balance equations. We then find analytical formulae for metastable and stable corona compositions. Finally, we present a reduced complexity model and sensitivity analysis, followed by numerical simulations. Table 1 lists the symbols used in this manuscript. We denote initial concentrations of nanoparticles and type 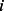 proteins as 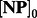 and 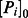, respectively. The concentration of bound type 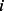 proteins in metastable and stable compositions are 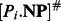 and 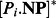, respectively.

**Table 1:** List of symbols used in this manuscript.

Symbol	Description
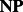	a nanoparticle
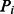	a free type 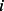 protein
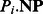	a type 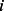 protein bound to a nanoparticle
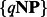	a corona complex with fraction 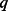 of its surface available for proteins binding
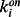	association rate of type 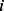 proteins to nanoparticles
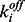	dissociation rate of type 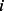 proteins from nanoparticles
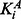	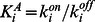 equilibrium constant of type 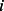 proteins,
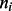	average number of type 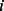 proteins fully covering a single nanoparticle
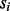	average surface fraction of nanoparticles occupied by type 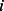 proteins

### Dynamical Model of Nanoparticle-Protein Corona Complex Formation

Here, we present a more comprehensive model for the evolution of protein concentrations than that of [1], given that ours is based on more descriptive biochemical equations. For comparison of Dell'Ocro *et al.* model with ours, please refer to the Discussion Section. Similar to Dell'Ocro *et al.* model [1], we only consider the two main processes that lead to corona formation, association and dissociation of proteins with nanoparticles.

Several other processes are important for corona formation process. For example, proteins can have a conformational change upon binding to a nanoparticle. In some cases, this conformational change is permanent [6]. Several researchers report irreversible bindings of blood proteins to nanoparticles [5], [15]. Similar to [1], we model only reversible bindings. The major extension to Dell'Ocro *et al.* model [1] is consideration of successive bindings to nanoparticles through more comprehensive biochemical equations. Importantly, we derive our dynamical model of corona complex formation process directly from corresponding population balance equations.

**Figure 1:**
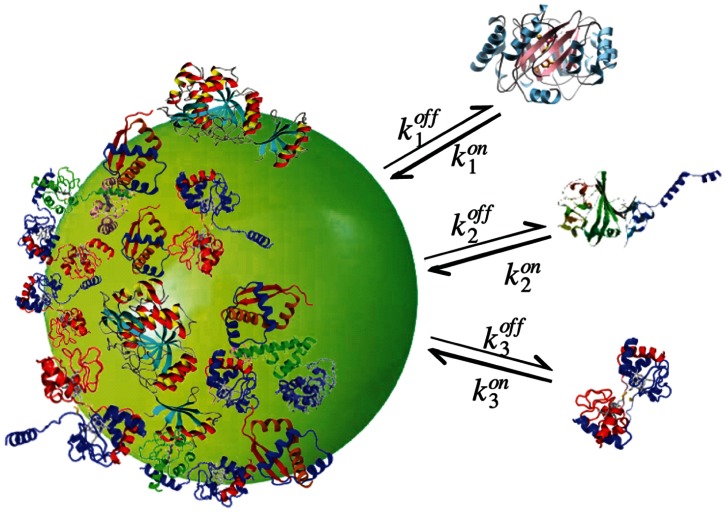
Schematic of nanoparticle-protein corona formation process. Single type 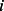 proteins attach to the nanoparticle surface at rate 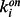, leaving the nanoparticle at rate 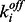. On average, a total number of 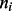 type 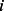 proteins can fully cover the nanoparticle surface.

For our modeling purpose, a protein or any other biomolecule is determined by three parameters: association rate 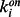, dissociation rate 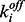, and the average number 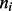 of type 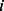 proteins fully covering a single nanoparticle. Figure 1 depicts a schematic of the corona formation process.

Assuming a corona complex has 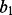 number of 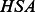 proteins, 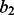 number of 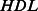 proteins, and 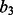 number of 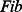 proteins bound to a nanoparticle, it can be denoted as 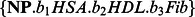. We describe the corona formation process in the following biochemical equations:
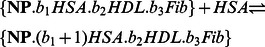
(1)

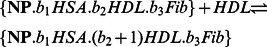
(2)

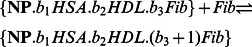
(3)


Challenges arise when solving corresponding population balance equations. First, the state space size is made enormous by too many possibilities for corona complex composition, depending on values of 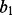, 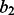, and 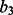. Second, it is perhaps impossible to find rate constants for the above transitions.

Recent studies (e.g. [17]) find proteins compete to bind to and spread across the curved surface of the nanoparticle, showing independent binding sites. Since there is evidence that protein-protein interactions are significantly weakened compared to nanoparticle-protein interactions due to the deflection angles on the convex surface of a nanoparticle [18], we ignore protein-protein interactions in modeling hard corona formation of spherical nanoparticles. For the binding process of a protein (e.g. 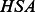) to a corona complex, these assumptions imply the free surface of the nanoparticle is paramount to the types and population of the proteins already associated with the corona complex. Accordingly, we can reformulate the equations by defining 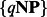 as a corona complex with fraction 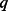 of its nanoparticle surface available for protein binding. We can then express the reversible biochemical equations describing the dynamics of the corona complex formation as
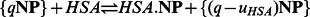
(4)

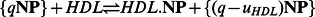
(5)

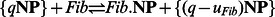
(6)


To illustrate the above equations, 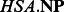 is an 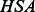 protein molecule bound to a nanoparticle. 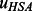, 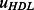, and 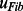 comprise the occupied surface fraction of the nanoparticle, given that a single protein binds to the nanoparticle. Since this fraction can vary depending on the geometrical configuration of the protein molecules, , 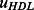, and 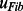 represent random variables assumed independent from each other. For example, biochemical equation (4) indicates that when a free protein 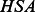 binds to a corona complex with fraction 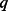 of the nanoparticle surface available for binding, the resulting corona complex has fraction 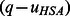 available for binding. The expected value for 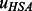, 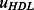, and 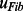 are 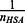, 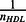, 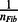, respectively, according to the definition of 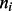. The average number of type 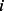 proteins that can fully cover a nanoparticle (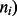 can be interpreted as the total number of nanoparticle surface binding sites available to type 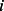 proteins. Therefore, we use a mass-action model to consider constant rates 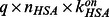, 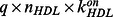, and 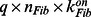, for association processes in biochemical equations (4)-(6), respectively.

Interestingly, population balance equations are tractable for the biochemical equations (4)-(6) despite the enormous state space of 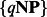 (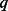 can take values between 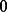 and 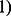. Regardless, the state space size for the protein molecules is 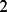 (protein molecules are either free or bound to nanoparticles), thus, allowing tractability of population balance equations of bound protein concentrations. In the Methods Section, the described derivation steps show concentration of bound proteins evolves according to the following ordinary differential equations:
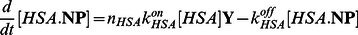
(7)

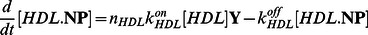
(8)

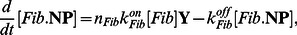
(9)where 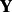 is defined as



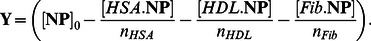
(10)In this paper, we consider a general case where the fluid contains 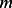 types of proteins interacting with nanoparticles. Similar to (7–10), the concentration 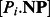 of type 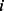 proteins bound to nanoparticles evolves as:
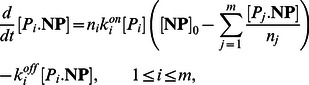
(11)where the following conservation laws holds for free protein concentrations:
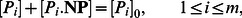
(12)because the sum of free and bound proteins is always equal to the initial concentration of proteins. We refer to (11) as the dynamical model of nanoparticle-protein corona complex formation.

Seeking expressions for biocorona complex composition, we define 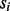 as the surface fraction of nanoparticle occupied by type 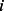 proteins. If 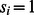, the nanoparticle surface is fully covered by type 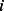 protein where 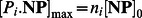. The surface fraction occupied by type 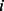 protein is the ratio between concentration 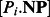 to 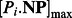:
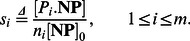
(13)


Because 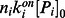 is much greater than 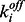, the dynamical system (11) exhibits two phases, an observation supported by experiments and numerical simulations [1], [3].

The fast phase corresponds to the transient time of system evolution during which surrounding proteins rapidly cover free nanoparticles. We refer to this stage as *metastable composition.* Corona complex composition changes after the initial phase, creating two distinct phases in the corona formation process. During the second phase, system evolution occurs at a much slower rate while bound proteins slowly leave nanoparticles and are potentially replaced by other proteins. We characterize the long run composition after the second phase as *stable composition*, which will not change at the macroscopic level, given an unchanging environment.

### Metastable Composition

As discussed earlier, nanoparticles are rapidly covered by proteins during the initial phase. We denote the metastable concentration of type 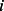 proteins bound to nanoparticles as 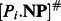. The type 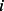 protein association process continues until there is either no more type 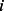 protein in the environment, i.e., 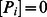, or nanoparticles are fully covered, i.e., 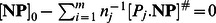. In this paper, we assume enough proteins in the environment to negate the first scenario. According to the model, proteins compete to attach to nanoparticles at rate 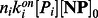 until the nanoparticles are fully covered. From this, we can derive an implicit solution for the exact value of 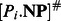. Since the corresponding algebraic equations are highly nonlinear and difficult to solve, we adopt an approximation proving compatible with simulation results:

Protein type 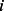 attaches to nanoparticles at rate 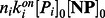 instead of 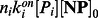. Using this approximation, metastable concentrations 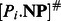 are
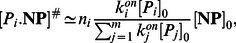
(14)for 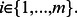 Expression (14) is a simple, well-structured formula that can be easily applied. To further simplify, this equation can be re-expressed as:



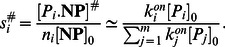
(15)The above equation enables the right hand side to function independently of 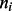 (i.e., the total number of binding sites for type 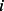 proteins). In (15), 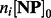 refers to the concentration of type 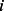 proteins if the nanoparticles are fully covered by type 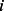 proteins. Therefore, 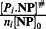 is the average surface fraction of the nanoparticle covered by type 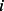 proteins.

### Stable Composition

The slow dynamics correspond to the dissociation of proteins from nanoparticles and subsequent replacement by other protein molecules. Setting the time derivative equal to zero in the differential equation (23), the stable composition of the corona complex is:
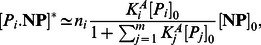
(16)where 
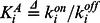
 is the *equilibrium constant* of type 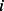 proteins. This equation can be re-expressed as:



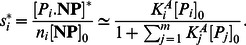
(17)Similar to (15), expression (17) exhibits independence from 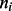. The stable composition formula (17) depends only on initial protein concentrations and equilibrium constants. For example, if a fluid contains only 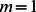 protein with initial concentration 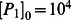 M and the equilibrium constant is 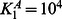 M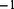, formula (17) predicts 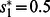 of surface coverage by proteins. This replicates the prediction in [6]. In practice the free area is very small, since 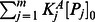 is normally much larger than 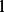.

### Reduced Complexity Model

As discussed earlier, the nanoparticle-protein corona formation model in (23) exhibits fast and slow dynamics. Therefore, numerical simulation of the model can be very time-consuming, requiring incremental time steps to guarantee numerical stability of simulations. We use techniques of nonlinear dynamics to decouple the fast and slow dynamics in the corona formation process. Using the singular perturbation technique found in the Materials and Methods Section, the evolution of the slow dynamic states 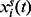 are described as:
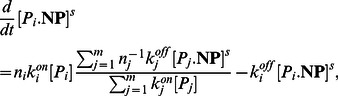
(18)where the initial condition is the metastable composition from (14). In this model, all terms are of the order of dissociation rates. Therefore, fast dynamics are omitted from the reduced complexity model to dramatically improve stability and run time of numerical simulations.

Using our reduced model, we observe the short run time of the original simulation is reduced nearly 1000 times for the 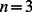 case. For 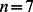 types of proteins, MATLAB® takes hours to solve the ODE system while the reduced system solves in a fraction of a second. Additionally, the reduced model does not require the absolute values of the parameters, 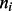, 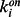, and 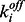, as ratio of these parameters is sufficient. Finding relative values is less error-prone since all parameters must be estimated through experiments. Significantly, our reduced model allows us to create discrete time scales. In fact, the Material and Method Section shows the time constant of the fast dynamics to be:
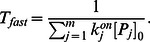
(19)


Therefore, the metastable state time constant 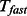 is of the order of the association rate inverses weighted by the initial protein concentrations. Moreover, (18) shows the stable state time constant is related to the inverse of the dissociation rates, implying the time constant is larger than the inverse of the smallest dissociation rate.

### Sensitivity Analysis

Sensitivity analysis of mathematical models is crucial in assessing reliability of predictions. In sensitivity analysis, we test how predictions of the model change with respect to a change or uncertainty in the involved parameters. If relying strictly on simulative results, we need to run several simulations for different values of the parameters in a combinatorial manner and compare outputs to understand sensitivity of the model to parameters. A very concrete advantage of our analytical results is the explicit information we derive from them.

To reduce complexity, the structure of our analytic formulae allows us to take the partial derivative of the formula related to influential parameters, thus obtaining analytical expressions of corona composition parameter sensitivity. Specifically, we perform a sensitivity analysis of model prediction for metastable and stable corona compositions respective to association rates and equilibrium constants by taking the partial derivative of 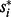 in (17) and 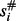 in (15) with respect to 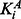 and 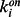, respectively, to obtain
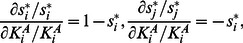
(20)

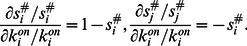
(21)


The above formulae indicate that relative changes in 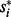 or 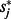 as the result of a relative change in 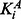 solely depend on the absolute value of 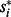. For example, if the stable composition formula (17) suggests 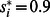, then a 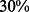 over-estimation of 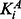 indicates an overestimation of 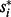 by nearly 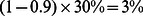 error.

### Numerical Simulations

While numerous types of biomolecules undergo hard corona formation, we follow [1] in considering only 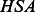, 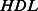, and 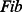 for numerical simulations. We use the initial concentrations values and corona formation parameters in Table 1 of [1].

**Figure 2:**
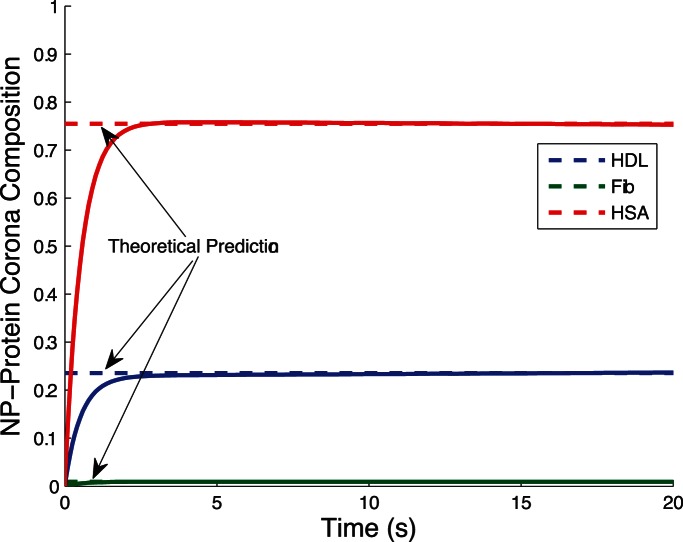
Initial phase of the corona formation process. During the initial phase, proteins rapidly cover the surface area of nanoparticles with rare occurrence of protein dissociation. A metastable composition of the corona complex results from this phase. In this simulation, the nanoparticle surface is covered by roughly 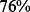 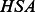 and 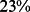 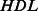 proteins. 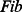 proteins constitute less than 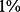 of the nanoparticle surface. Significantly, there is excellent agreement between the theoretical predictions from our metastable composition formula (15) and the numerical simulation results.

**Figure 3:**
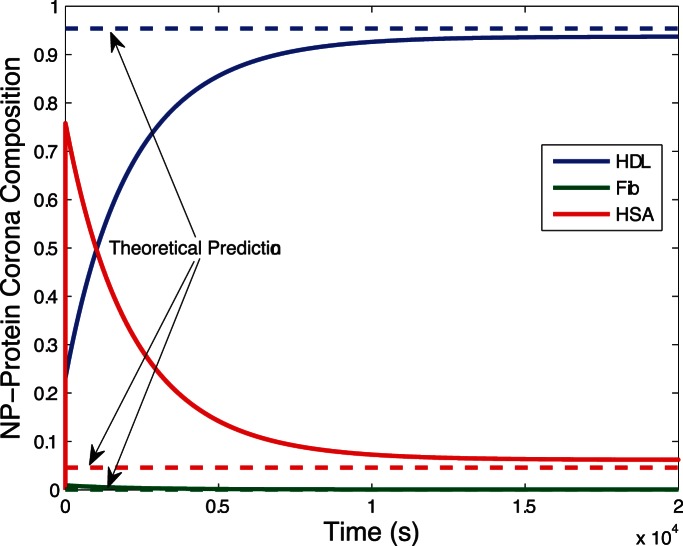
Long run simulation of corona formation process. The initial metastable composition of the corona complex fluctuates from dissociation and replacement of proteins from nanoparticles. The final composition of the corona complex is stable, given the environment does not change. In this simulation, about 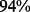 of the nanoparticle surface is covered by 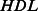 proteins and about 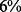 is covered by 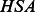 proteins, with a negligible amount of 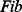 proteins. Again, there is strong agreement between the theoretical predictions from the stable composition formula (17) and the numerical simulation results.

Figure 2 shows the evolution of biocorona composition during the initial phase of the corona formation process. Dissociation of proteins from nanoparticles rarely occurs during this stage. After the initial phase where proteins surround the nanoparticle, the corona composition changes as proteins dissociate from nanoparticles and are possibly replaced by other proteins. The evolution of biocorona composition is plotted in Figure 3. Figures 2 and 3 show theoretical predictions for metastable (15) and stable (17) corona complex compositions agree with numerical simulations.

**Figure 4:**
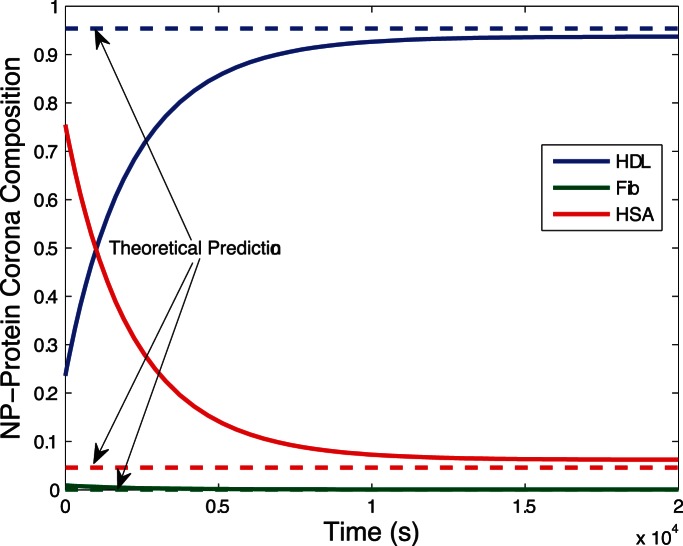
The response of the reduced complexity corona formation model (18) representing slow dynamics of the corona formation process. The initial condition for the reduced complexity model is the metastable corona composition from (14). The response of the reduced complexity model (18) is in excellent agreement with the response of the original model (11) shown in Figure 3, except for the short initial behavior that belongs to the fast dynamics of the system evolution.

Fast and slow dynamics in the original corona formation model makes numerical solutions very time consuming. The reduced complexity model (18) captures the response of slow dynamic, dramatically reducing numerical simulation run time and improving stability. Figure 4 shows the response of the reduced model (18) accurately reproduces the response of the original model (11) with added capability of robust, rapid numerical simulation.

**Figure 5:**
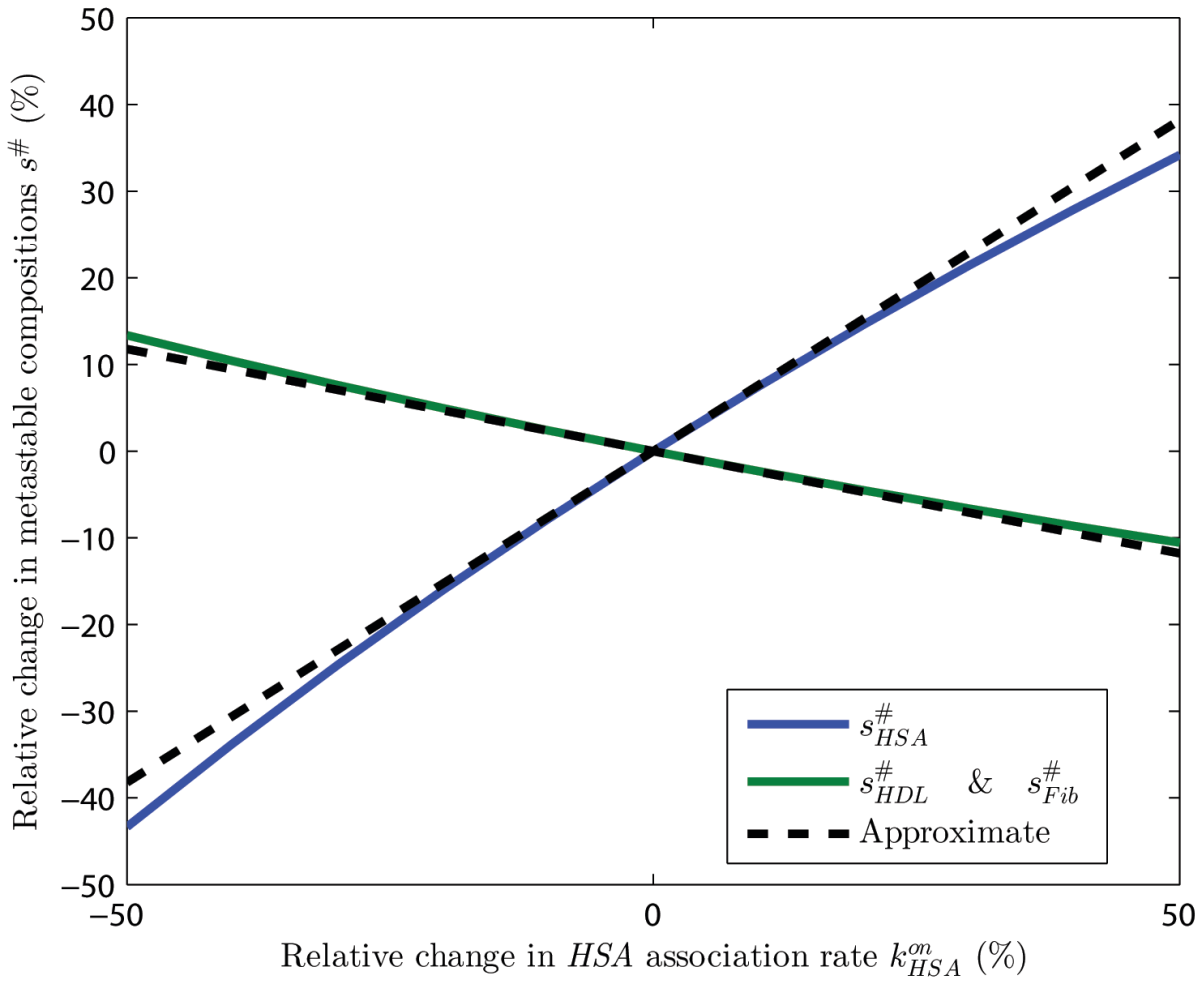
Sensitivity of metastable corona composition incorporating uncertainties in association rate of HSA proteins. Derivative with respect to 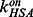 reasonably interprets the true relative change of composition. Overestimating 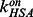 results in overestimation of 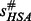 and underestimation of 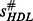 and 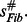

We perform a numerical study of the metastable corona composition sensitivity incorporating 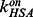, letting 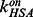 deviate from its nominal value up to 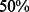. We then calculate relative change in metastable composition from (15). Figure 5 shows increasing the association rate 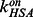 obtains higher composition fraction for 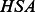 proteins, the opposite being true for 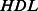 and 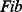 proteins. For example, a 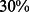 increase in 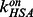 increases 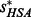 (blue line) 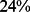 while 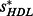 and 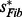
(green line) decrease 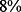. The local sensitivity analysis (20) provides sound understanding of sensitivity up to a drastic 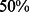 change.

## Discussion

In this paper, we extend the modeling and simulation results in [1] with relevant analytical results. The model provides a simplified representation of the complex process of corona formation yet quantitatively justifies experimentally supported observations that corona complex composition rapidly reaches metastable equilibrium to slowly converge towards a stable composition. We obtained two simple and expressive formulae, (15) and (17), providing the composition of the nanoparticle corona in the metastable and stable states. These formulae mathematically predict the corona composition free of simulations through insertion of appropriate parameter values. Figures 2 and 3 prove our mathematical predictions of the metastable and stable corona compositions are very accurate compared to simulations. Moreover, the composition formulae (15) and (17) afford easy computation, providing direct insight into the key parameters of each phase of corona formation process.

Viewed against Dell'Ocro *et al.* model [1], ours (see (7–9)) replicate nearly all of the their results except their drastically shortened time constant of metastable equilibrium. Significantly, Dell'Ocro *et al.* model interprets other features of the corona complex formation dynamics, including the stable and metastable compositions resulting from the law of large numbers, and the slow dynamics time constant having the order of dissociation rate inverses [1]. Our markedly lengthening of metastable equilibrium time constant is due to our expansion of Dell'Orco *et al.* consideration of the following nanoparticle-protein binding equations:
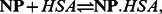


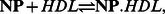


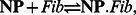
to biochemical equations (1–3). Relatively speaking, our proposed biochemical equations (1–3) better describe dynamical evolution of corona composition in considering *successive bindings* of protein molecules to a nanoparticle, resulting in a much longer time constant for initial transient dynamics.

Composition of the nanoparticle corona can derail the nanoparticle mission from therapeutic and beneficial to toxic and dangerous. The benefit of the proposed results for scientists in this field is multifaceted: these formulae guide experimentation and aid interpretation of experimental results, increasing knowledge of *in vivo* nanoparticles behavior. Analytical results derived from (15), (17), (20), and (21) affirm assessment and standardization of critical behaviors of nanoparticles in body fluids or any other *in vivo* or *in vitro* environments, showing promising medical and therapeutic applications.

We understand that the model in [1], and consequently our model, describe a simplified system. For example, we only consider nanoparticles with sphericity close to 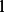. Nonetheless, our results can help develop more accurate models, guide the selection of specific sets of experiments, and ultimately increase knowledge of the corona complex formation dynamics. Consideration of more detailed aspects such as, soft corona formation, protein-protein interactions, irreversible bindings, conformational change of proteins, and persistent stochastic fluctuations, demands further modeling work.

## Methods

Below, we detail the derivations of analytical results found in the Results Section.

### Derivation of Dynamical Model for Corona Complex Formation

In this section, we demonstrate derivation of the dynamical model (7–9) for the corona complex formation from the biochemical equations (4–6).

The population balance equations for the biochemical equations (4–6) suggest the concentration of proteins bound to a nanoparticle (e.g. 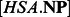) evolves according to the following differential equation:
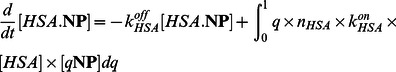


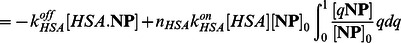



If the number of nanoparticles is large, 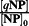 denotes the probability that the free surface fraction of a nanoparticle is equal to 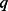. Therefore, 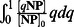 in fact represents the expected value of 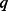, i.e.,
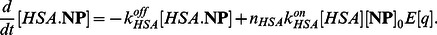



The free surface fraction is one minus the occupied surface fraction, i.e., 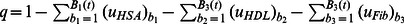 where, for example, 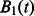 is the number of 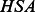 proteins bound to the nanoparticle. Since expectation of a random variable is a linear operator, we derive:
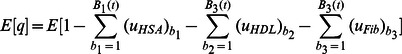


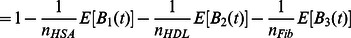


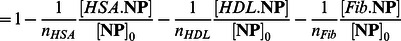


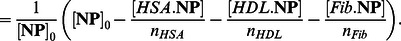



Hence, we obtain the dynamical model (7–9) for corona complex formation. MATLAB® version R2010a was used to simulate the ordinary differential equations of corona formation dynamical model.

### Stable Composition Formulae

In this section, we obtain the formulae for the stable composition of the corona complex in (16). To facilitate subsequent analysis, we define the system state 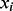 as the concentration of type 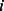 proteins bound to nanoparticles:
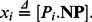
(22)


Incorporating the conservation law (12), evolution of bound type 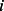 proteins concentration 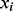 is described:
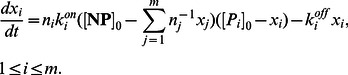
(23)


For a nonlinear dynamic system [19] following the differential equation 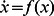, the equilibrium points are the solutions of algebraic equation 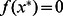. Setting the time derivatives equal to zero in the differential equation (23), the equilibrium equations satisfy:
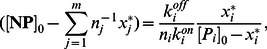
(24)for 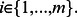. Therefore, 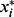 can be written as:



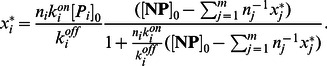
(25)Exact solution of the above equations is very difficult and complicated. However, we can approximate (25) as:
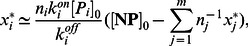
(26)knowing that the total nanoparticle surface is occupied by proteins, thus making 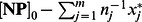 very small. Hence, from (26),
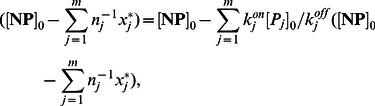
(27)from which 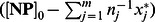 is obtained as



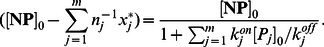
(28)Replacing for 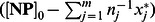 in (26) yields
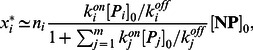
(29)which is (16).

### Derivation of Reduced Complexity Model

In this section, we provide details for obtaining the reduced complexity model in (18). The model reduction technique separating the fast and slow dynamics is the topic of singular perturbation method. Specifically, a dynamical system
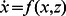


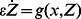
with small scalar 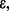 can be approximated by a system of the form 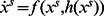, where 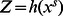 is the solution of 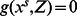. Those readers interested in learning more about these nonlinear dynamics techniques are referred to the classical textbook [19].

First, we define the auxiliary state 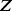 as:
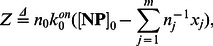
(30)where 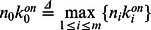. According to (23) we have



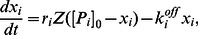
(31)

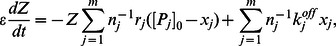


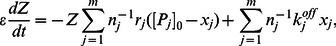
(32) where 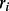 and 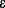 are



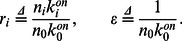
(33)Because the system (31)-(32) has the standard singular perturbation form, we use the singular perturbation technique to find the slow dynamics. According to (32), the equilibrium value of 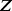 is
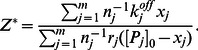
(34)


Singular perturbation technique replaces the value of 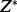 with 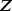 in (31). Therefore, the evolution of the slow dynamic states 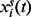 is described by:
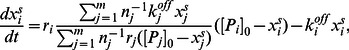
(35)which leads to (18). From (32), the time constant of the fast dynamics is
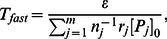
(36)leading to (19).
